# Frailty in Glioblastoma Is Independent From Chronological Age

**DOI:** 10.3389/fneur.2021.777120

**Published:** 2021-11-30

**Authors:** Harald Krenzlin, Dragan Jankovic, Christoph Alberter, Darius Kalasauskas, Christiane Westphalen, Florian Ringel, Naureen Keric

**Affiliations:** ^1^Department of Neurosurgery, University Hospital Mainz, Mainz, Germany; ^2^Department of Geriatrics, University Hospital Mainz, Mainz, Germany

**Keywords:** glioblastoma, frailty, Groningen Frailty Index, G8, geriatric patients

## Abstract

**Objective:** Treatment of glioblastoma in elderly patients is particularly challenging due to their general condition and comorbidities. Treatment decisions are often based on chronological age. Frailty screening tests promise an assessment tool to stratify geriatric patients and identify those at risk for an unfavorable outcome. This study aims to evaluate the impact of age and frailty on the surgical outcome and overall survival in geriatric patients with glioblastoma.

**Methods:** Data acquisition was conducted as a single-center retrospective analysis. From January 1st 2015, and December 31st 2019, 104 glioblastoma patients over 70 years of age were included in our study. Demographic data, tumor size, Karnofsky Performance Score (KPS), and Eastern Cooperative Oncology Group Performance Status (ECOG), as well as treatment modalities, were assessed. The Geriatric 8 health status screening tool (G8) and Groningen Frailty Index (GFI) were compiled pre-and postoperatively.

**Results:** The mean patient age was 76.86 ± 4.11 years. Forty-nine (47%) patients were female, 55 (53%) male. Sixty-seven patients underwent microsurgical tumor resection, 37 received tumor biopsy alone. Mean G8 on admission was 12.4 ± 2.0, mean GFI 5.0 ± 2.5. In our cohort, frailty was independent of patient age, tumor size, or localization. Frailty, defined by G8 and GFI, is associated with shorter overall survival (G8: *p* = 0.0035; GFI: *p* = 0.0136) and higher numbers of surgical complications (G8: *p* = 0.0326; GFI: *p* = 0.0388). Frailer patients are more likely to receive best supportive care (*p* = 0.004). Nevertheless, frailty did not affect adjuvant treatment decision-making toward either single-use of chemo- or radiation therapy, stratified treatment, or concomitant therapy. The surgical decision on the extent of resection was not based on pre-operative frailty.

**Conclusion:** In our study, frailty is a predictor of poorer surgical outcomes, post-operative complications, and impaired overall survival independent of chronological age. Frailty screening tests offer an additional assessment tool to stratify geriatric patients with glioblastoma and identify those at risk for a detrimental outcome and thus should be implemented in therapeutic decision making.

## Introduction

Glioblastoma is the most common primary malignant brain tumor in adults with a dismal prognosis (1). Population studies have shown that survival declines with increasing age while the incidence increases, especially among the elderly over 70 years (2, 3). As of today, no unanimous definition of when a patient is defined as an “elderly” exists. The WHO propagates an age limit of 60–65 years, although the prevalence of age-defining symptoms such as loss of hearing, impaired vision, sleeplessness, incontinence, and physical and mental deterioration start to increase in patients 70–75 years (4). Given the poor overall prognosis, frequent coexisting conditions, and an increased risk of toxic effects from chemo- and radiotherapy on the aging brain, glioblastoma management in patients 65 years or older is exceedingly complex (5). Progress has been limited for decades, as clinical trials traditionally used upper age limits excluding elderly patients (6). Older patients with glioblastoma have been underrepresented in clinical trials, as the average age of participants is 55 years compared to 65 years in population-based studies (3). Recently, randomized data for the treatment of elderly patients with glioblastoma has been provided by trials conducted by the Scandinavian Neuro Oncology Network, the Neuro oncology Working Group of the German Cancer Society (NOA), as well as the Canadian Cancer Trials Group (CCTG) and the European Organization for Research and Treatment of Cancer (EORTC) (6–8). Evidence supports maximal safe surgical resection, the superiority of the concurrent radio-chemotherapy compared to TMZ or radiotherapy alone, and equivalency of short-course radiotherapy compared to longer treatments (8, 9). These studies establish a new paradigm for treating elderly patients over 65 years. Nevertheless, across-the-board treatment decisions based on chronological age are no longer feasible in the context of individualized medicine. Old age alone is not associated with increased perioperative complication rates, such as infections, prolonged intensive care treatment, and slower recovery (10). A growing body of evidence suggests that frailty is a more appropriate predictor of surgical outcome, post-operative complications, and impaired overall survival than chronological age (11). Although frailty screening tests offer assessment tools to stratify geriatric patients and identify those at risk for a detrimental outcome, they are not commonly used in informing surgical decisions (12).

This study aims to evaluate the impact of age and frailty measured using the Groningen Frailty Index (GFI) and the G8 questionnaire on the surgical outcome and long-term survival in geriatric patients with glioblastoma.

## Patients and Methods

### Patients

All patients over the age of 70 with newly diagnosed glioblastoma treated at our hospital between January 1st 2015, and December 31st 2019, were included in our study. Baseline characteristics, including age, sex, functional neurological status at admission and discharge, as well as radiological and molecular tumor features, were recorded. The Karnofsky performance score (KPS), the ECOG (Eastern Cooperative Oncology Group) performance status, the Groningen frailty index, and the G8 Questionnaire were used to evaluate geriatric patients according to their frailty and functional status. All patients either received stereotactic biopsy or tumor resection. Early (<72 h) post-operative magnetic resonance imaging (MRI) was used to determine the extent of resection. Complete resection of the contrast-enhancing tumor was deemed gross-total resection (GTR). Progression free survival (PFS) and overall survival (OS) measured in weeks were defined from surgery until radiological progression or death, respectively. During institutional interdisciplinary tumor board meetings, treatment decisions concerning the surgical procedure and adjuvant treatment were made prior to and after surgery.

### The Groningen Frailty Index and the G8 Questionnaire

The GFI is a 15-item questionnaire with a score range from zero to fifteen. Four principal dimensions, physical, cognitive, social, and psychological, are assessed. A score of four or greater is considered as a cut-off point for frailty (13). The G8 questionnaire is a screening tool for comprehensive geriatric assessment (CGA) in elderly oncological patients. It consists of seven questions plus age. The cut-off value for identifying frailty in cancer patients with the G8 questionnaire has been previously determined as 12.5 (AUC of 0.87 (95% CI: 0.81–0.92; SE 0.03) (14, 15) (Supplementary Materials 1, 2).

### Statistics

Data analysis was performed using the computer software package SPSS (version 25, IBM Corp., Armonk, NY). Unpaired categorical and binary variables were analyzed in contingency tables using Fisher's exact test. The Mann–Whitney U-test was chosen to compare continuous variables as the data were mainly not normally distributed. OS was analyzed by the Kaplan–Meier method using Gehan–Breslow–Wilcoxon test. The hazard ratio was calculated using the Mantel-Haenszel test. Results with *p* < 0.05 were considered statistically significant. Finally, a backward stepwise method was used to construct a multivariate logistic regression model to validate age, ECOG, KPS, G8, GFI, MGMT, and resection as predictors of PFS and OS.

### Ethical Approval

Data acquisition and analysis were performed anonymously and were approved by the Ethics Committees of the medical association of Rhineland Palatinate, Germany. According to the local laws, no informed consent is necessary for such kind of retrospective analysis.

## Results

### Baseline Characteristics

Between January 1st 2015 and December 31st 2019, 104 consecutive patients aged 70 years or older with newly diagnosed Glioblastoma were treated at our department. Of all patients, 49 were female, 55 male. The patient's age ranged from 70 to 89 years (76.60 ± 4.41). Between all patients, median pre-operative KPS was 70 (range 30–100), mean ECOG was two (range 0–4). At the time of discharge, the mean KPS was 85 (range 30–100), mean ECOG was two (range 0–4). On admission, geriatric patients with glioblastoma had a median GFI of five (range 1–11) and a median G8 score of 12 (6–15). According to GFI, 43 (41.34%) geriatric patients with glioblastoma showed no signs of frailty, 51 (49.04%) according to the G8 Questionnaire. Tumors most frequently involved the temporal lobe (39.4%), followed by the frontal (25.9%), parietal (18.3%), and occipital (3.8%) lobe, and deeper regions (12.5%). In 14 patients (13.5%), the tumor involved both hemispheres. Biopsy was performed in 36 patients (35.6%), GTR in 45 (43.3%) and STR in 22 (21.2%). Methylation of the MGMT promotor was detected in 46 patients (44.23%). All tumors were IDH 1/2 wild-type. Median OS was 29 weeks (95% CI 22.9–35.8) (Table 1).

**Table 1:** Baseline demographics and clinical characteristics.

	**Entire cohort** **(*n* = 104)**	**Not frail patients** **(*n* = 36)**	**Frail patients** **(*n* = 68)**
**Gender**			
Female	49 (47%)	13 (12.5%; ns)	36 (34.5%; ns)
Male	55 (53%)	23 (22%; ns)	32 (31%; ns)
**Age** (+SD)	76.60 ± 4.41	76.69 ± 4.66 (ns)	76.42 ± 3.945 (ns)
**Tumor size**	35.43 ± 18.90	30.00 ± 14.17 (ns)	38.30 ± 20.49 (ns)
**ECOG**			
Admission	1.77 ± 0.99	1.25 ± 0.87 (ns)	2.04 ± 0.94 (ns)
Discharge	2.15 ± 1.12	1.47 ± 0.99 (^****^)	2.51 ± 1.00 (^****^)
**KPS**			
Admission	70.7 ± 13.5	78.1 ± 10.6 (ns)	66.8 ± 13.3 (ns)
Discharge	85.44 ± 0.23	94.44 ± 0.23 (ns)	80.6 ± 0.39 (ns)
**MGMT**			
Methylated	46 (44%; ns)	16 (44%; ns)	30 (44%; ns)
Unmethylated	58 (56%; ns)	20 (56%; ns)	38 (56%; ns)
**Resection**			
GTR	66 (63.5%; ns)	28 (80%; ns)	38 (56.7%; ns)
PR/Biopsy	36 (34.6%; ns)	7 (20%; ns)	29 (43.3%; ns)
**Radiation**			
Definitive	24 (27%; ns)	12 (42%; ns)	12 (20%; ns)
Concomitant	17 (19%; ns)	9 (32%; ns)	8 (13%; ns)
**Chemotherapy**	20 (22%; ns)	5 (18%; ns)	15 (25%; ns)
**Best supportive care**	28 (31%; ns)	2 (7%; ns)	26 (43%; ns)

*ns, not significant*;

**** *p < 0.001*.

### Frailty Is Independent of Chronological Age and Concomitant Comorbidities

**Figure 1:**
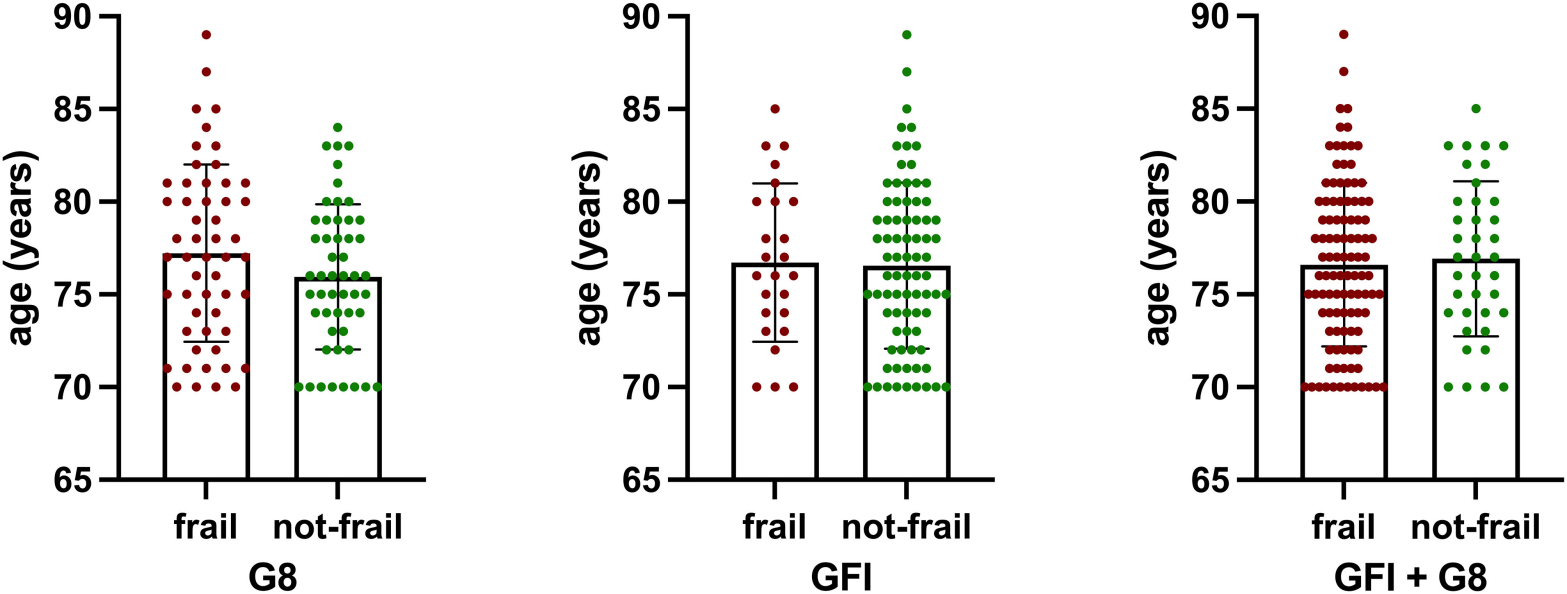
There was no statistically significant difference in age of patients defined as frail by either metric (G8, *p* = 0.1379; GFI, *p* = 0.8729) or a combination of both (G8 + GFI, *p* = 0.6940).

Patients defined as frail by the G8 score had a mean age of 77.2 ± 4.8 years; those defined as not-frail were 75.9 ± 3.9 years (*p* = 0.1379). According to GFI, frailer patients were 76.7 ± 4.3 years old, compared to 76.6 ± 4.5 years (*p* = 0.8729). Frail patients, according to either metric, were 76.6 ± 4.4 years of age, those not-frail 76.9 ± 4.2 (*p* = 0.6940) (Figure 1). The mean CCI of patients defined as frail was 7.906 ± 1.061 (G8, *p* = 0.9088) and 7.905 ± 0.982 (GFI) (*p* = 0.9486). All patients had a CCI of six and higher.

### Frailty Is Associated With Shortened Overall Survival

**Figure 2:**
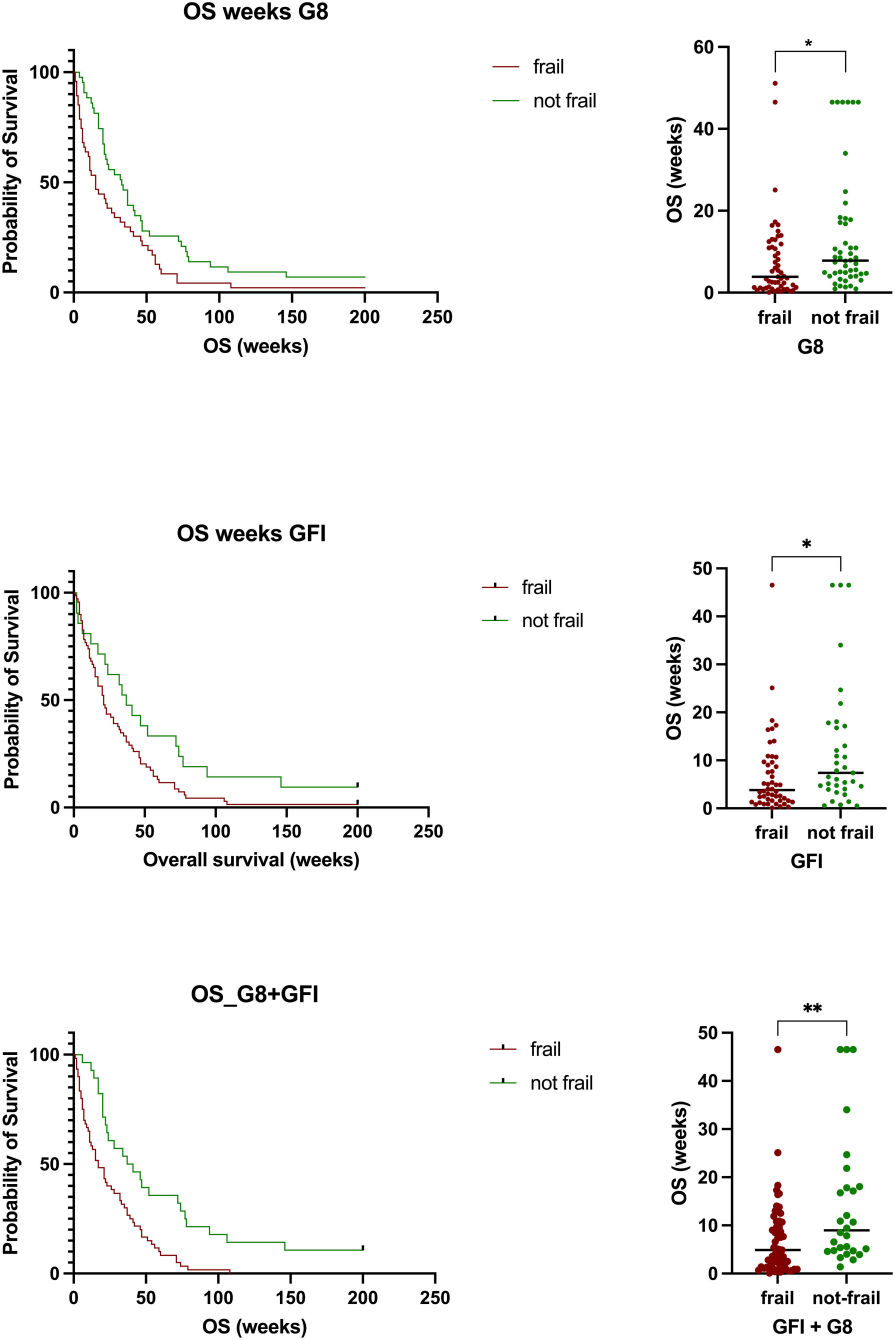
Patients defined as frail by either scale or a combination of both had a statistically significant shorter overall survival compared to those defined as not frail (G8, *p* = 0.0216; GFI, *p* = 0.0167) or a combination of both (G8 + GFI, *p* = 0.0025).

According to the G8 score, 53 patients were defined as frail, compared to 61 using the GFI and 69 using a combination of both scales. Geriatric patients with glioblastoma defined as frail according to the G8 questionnaire had a median OS of 7.7 ± 10.1 months. In comparison, not-frail patients had a median OS of 13.4 ± 14.3 months (*p* = 0.0216). Patients defined as frail according to the GFI had a median OS of 6.7 ± 8.1 months. Those defined as not-frail had a median OS of 12.3 ± 13.0 months (*p* = 0.0167). Patients defined by both metrics as frail had a median OS of 7.1 ± 7.8 compared to 14.3 ± 13.7 months in those defined as not-frail (*p* = 0.0025) (Figure 2). Survival analysis revealed a statistically significant shorter survival in frail patients with glioblastoma according to the G8 questionnaire (HR = 1.743, 95% CI 1.121–2.711, *p* = 0.0136) as well as the GFI (HR = 1.672, 95% CI 1.087–2.570, *p* = 0.0035) and those patients classified as frail with either G8 or GFI (HR = 2.272, 95% CI 1.448–3.563, *p* = 0.0004) (Figure 2).

### Frailty and Post-operative Morbidity

**Figure 3:**
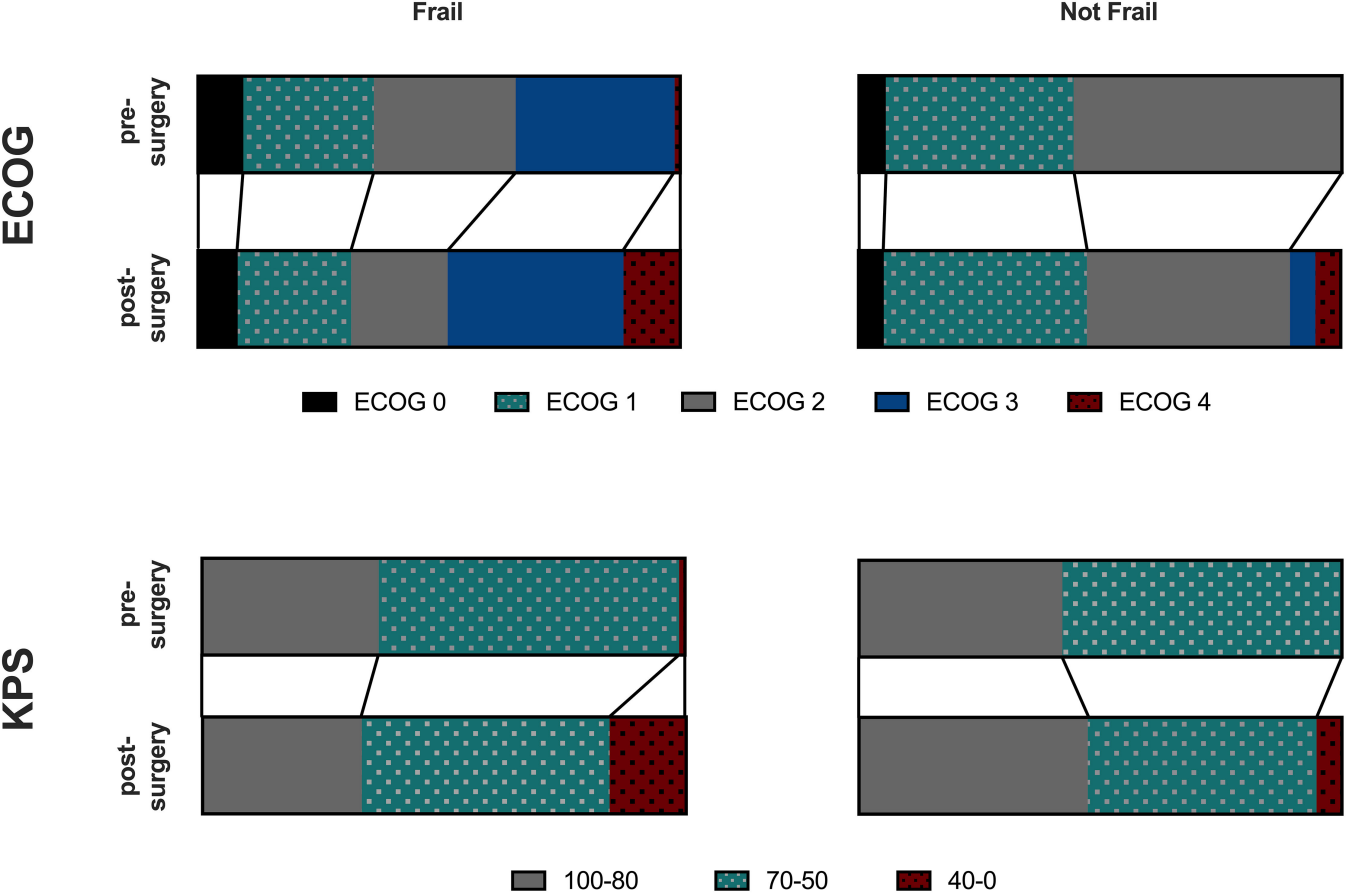
ECOG or KPS was similar in frail and not frail Patients. Those defined as frail had a higher likelihood of developing postsurgical complications and post-operative ECOG status was significantly worse in frail patients using either of the two scales using the G8 (*p* < 0.0001), GFI (*p* < 0.0001) or a combination of both (*p* < 0.0001).

Geriatric patients had a higher likelihood of developing post-surgical complications if identified as frail using the G8 (OR = 3.6795, 95% CI 1.1143–12.1502, *p* = 0.0326), the GFI (OR = 4.0, 95% CI 1.0741–14.8961, *p* = 0.0388), or the combination of both (OR = 3.913, 95% CI 1.0515–14.5620, *p* = 0.0419). Pre-operative ECOG or KPS was similar in both groups. However, post-operative ECOG status (GFI: *p* < 0.0001; G8: *p* < 0.0001) and KPS (GFI: *p* < 0.0001; G8: *p* < 0.0001) was significantly worse in frail patients using either of the two scales (Figure 3). No difference was found between patients defined as frail/not frail by G8 or GFI.

### Treatment Data

**Figure 4:**
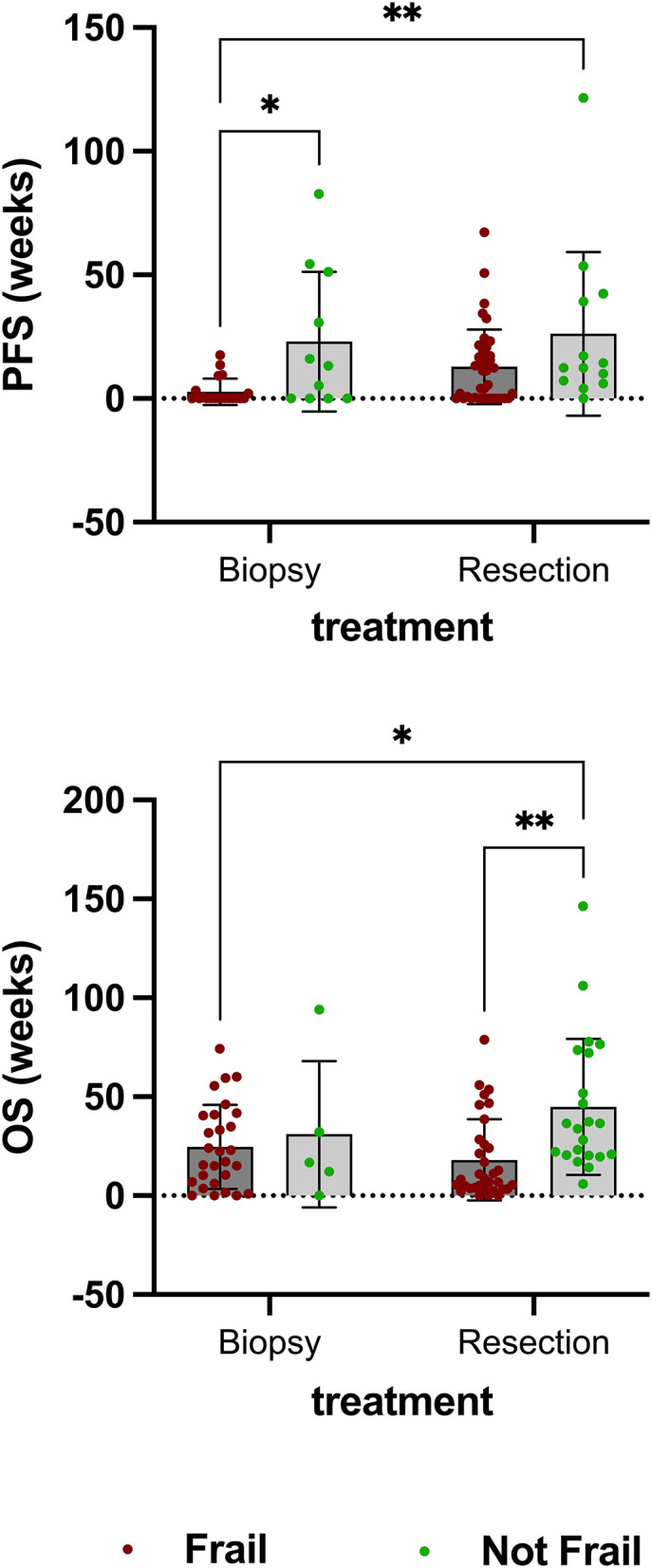
There was no statistically significant difference in the number (cases) of resections performed in patients stratified as not frail (75.00%) and in those defined as frail (58.73%). While tumor resection led to improved PFS in patients defined as frail compared to biopsy alone (*p* = 0.0069), it was only associated with improved overall survival in patients defined as not frail (*p* = 0.0017).

There was no statistically significant difference in the number (cases) of resections performed in patients stratified as not frail (75.00%) and in those defined as frail (58.73%). While tumor resection led to improved PFS in patients defined as frail compared to biopsy alone (*p* = 0.0069), it was only associated with improved overall survival in patients defined as not frail (*p* = 0.0017) (Figure 4). No statistically significant differences in OS were found between either frail or not frail patients treated with chemotherapy or radiation alone compared to a combination of both.

### Multivariate Analysis

A multivariate logistic regression analysis was conducted to identify independent predictors of OS in geriatric patients with glioblastoma. ECOG three (*p* = 0.028, OR = 2.520, 95% CI 1.106–5.741), radiotherapy (*p* = 0.026, OR = 2.219, 95% CI 1.1–4.47) and frailty detected by GFI (*p* = 0.017, OR = 0.895, 95% CI 0.818–0.980) were significant and independent predictors of OS. Age (*p* = 0.855, OR = 1.043, 95% CI 0.667–1.628), KPS (*p* = 0.320, OR = 0.530, 95% CI 0.131–2.142), MGMT methylation (*p* = 0.888, OR = 0.969, 95% CI 0.628–1.495) and extent of resection (GTR: *p* = 0.599, OR = 0.822, 95% CI 0.551–1.411; PR: *p* = 0.555, OR = 1.2, 95% CI 0.654–2.201) were no independent predictors of OS in geriatric patients with glioblastoma.

## Discussion

Demographic changes with an increased life expectancy led to a rapidly growing geriatric population. As high-grade gliomas are the most common central nervous system malignancy and are mostly diagnosed at a median age of 64 years, the incidence increases with growing life expectancy (2, 3). At the same time, the treatment of glioblastoma in elderly patients is particularly challenging due to their general condition and comorbidities (16). As of today, clinical data in geriatric patients with glioblastoma is scarce. Here, we evaluated patients over 70 years of age with newly diagnosed glioblastoma for the influence of age and prevalent frailty on surgical outcome and overall survival.

Currently, most treatment decisions are based on chronological age (17). The landmark study of Stupp et al. showed a benefit of radiotherapy plus temozolomide followed by adjuvant temozolomide to treat glioblastoma (18). However, only patients younger than 70 years were included in this trial. The addition of temozolomide has been shown to be less effective in patients between 65 and 70 years (19). Underrepresentation of elderly patients in clinical cancer trials leads to heterogeneous data on treatment effectiveness, as well as inconsistent and highly subjective treatment decision-making in this ever-growing group of patients. Older patients are often treated less aggressively due to a perceived lack of physical resilience in response to post-operative complications and treatment toxicity (20, 21). Our cohort reflects this circumstance as only a fraction has been treated concomitantly, while most received either adjuvant radiation or chemotherapy alone. The influence of the extent of resection on overall survival is still a matter of debate. The EORTC 26,062 trial showed the patients with tumor resection had significantly longer survival than those with biopsy only (8). Similar findings were reported in a randomized trial in patients older than 65 (22). The small number of patients severely hampered the clinical implication. Our study adds proof to this observation as we found that OS improved in patients receiving tumor resection compared to biopsy taking independent from preexisting frailty. As expected, patients undergoing resection had a higher likelihood of an improved neurological outcome, while those receiving biopsy alone remained unchanged or deteriorated.

Patients' frailty and comorbidity burden have recently emerged as predictors of morbidity and mortality in various types of cancer in older patients (14). This observation falls in line with the results of our study where patients identified as frail using either the G8 questionnaire, the GFI, or a combination of both have a significantly reduced overall survival. As patients over 70 years of age are underrepresented in clinical trials, there is even less data on the impact of chronological age in geriatric patients with different glioblastoma (6). Our data suggest that this void might be overcome by adding frailty as an additional marker to stratify older patients for those with favorable or unfavorable outcome as frailty is associated with the occurrence of surgical complications and shortened OS. In the present study, frailty has been assessed using the G8 and the GFI. Both instruments are capable of separating older patients with cancer according to their preexisting frailty. The G8 is supposed to offer a better sensitivity with less specificity compared to the GFI (14). Consequently, the combination of both scales provided the best results in identifying frailty in older patients with glioblastoma in our patients. Subsequently, increased frailty resulted in a significantly higher probability of poorer survival. In our highly selective cohort of patients, including only geriatric patients older than 70, chronological age was no longer a predictor of morbidity or overall survival in a multivariate analysis. This finding might argue in favor of a more stratified treatment approach as age alone might not suffice for informed decision-making in geriatric patients with glioblastoma. In our elderly patient collective, ECOG and KPS were no striking predictors of an individual outcome but improved after tumor resection, if the patient was not frail. Individual frailty and comorbidity burden might identify those patients with sufficient resilience for more intense treatment protocols and thus longer OS.

Modified treatment regimens have been proposed to minimize treatment-associated toxicity and adverse events in elderly patients with glioblastoma. Short course radiotherapy (34Gy for two weeks) proved to be as effective as standard radiotherapy (60Gy for 6 weeks) in patients older than 70 years (23). There is also evidence that temozolomide alone might be more efficient than radiotherapy in patients with methylation of the O6-methylguanine–DNA methyltransferase (MGMT) gene promotor region in the elderly (23). The combination of temozolomide and short-course radiotherapy resulted in a more prolonged survival than short-course radiotherapy alone in a large clinical trial funded by the Canadian Cancer Society Research Institute (8). Our data argues the same way as a combined treatment showed a tendency to prolonged PFS and OS without being statistically significant. As expected, frail patients seem to benefit less from adjuvant treatment, compared to those classified as not frail. Fittingly, best supportive care showed a similar PFS and OS in frail patients compared to all other treatment regiments. Applying a multivariate analysis, radiotherapy emerged as an independent predictor of OS in our patient cohort. However, the MGMT-promotor methylation and MGMT stratified treatment showed an inclination to prolonged OS without reaching statistically significance in our cohort.

However, there are several important limitations to our study. Due to a limited and heterogeneous group of patients, the influence of different therapeutic regiments on PFS and mOS might have been underestimated. Further bias might arise from involuntarily accounting for poor general health and signs of frailty during the process of treatment decision making. Rretrospective data collection and a lack of randomization are important limitation the generalizability of our study. To account for these shortcomings, future prospective studies with an increased number of patients and data acquisition sites might be capable to establish frailty not only as an important influence on PFS/mOS, but as an independent outcome predictor and parameter in the treatment of not only geriatric patients with glioblastoma.

## Conclusion

In our study, frailty is associated with a shortened overall survival in geriatric patients with glioblastoma. Thus, frailty screening is an essential and telling addition to clinical and demographical patient evaluation offering the possibility to improve the selection of suitable patients for different treatment strategies. Additionally, frailty screening provides insightful information to ameliorate counseling those patients and their families.

## Data Availability Statement

The original contributions presented in the study are included in the article/Supplementary Material, further inquiries can be directed to the corresponding author.

## Author Contributions

HK, DJ, CW, and NK contributed to conception and design of the study. CA and DJ organized the database. HK and DK performed the statistical analysis. HK wrote the first draft of the manuscript. FR and NK supervised the study. All authors contributed to manuscript revision, read, and approved the submitted version.

## Conflict of Interest

The authors declare that the research was conducted in the absence of any commercial or financial relationships that could be construed as a potential conflict of interest.

## Publisher's Note

All claims expressed in this article are solely those of the authors and do not necessarily represent those of their affiliated organizations, or those of the publisher, the editors and the reviewers. Any product that may be evaluated in this article, or claim that may be made by its manufacturer, is not guaranteed or endorsed by the publisher.
