# Supplementary material for: Frailty in Glioblastoma Is Independent From Chronological Age
Source: Front Neurol. 2021 Nov 30;12:777120. doi: 10.3389/fneur.2021.777120 (PMC8669893; doi:10.3389/fneur.2021.777120)
Supplement: Supplementary file 1 [file Data_Sheet_1.PDF]

Supplement 1)

**GFI (Groningen Frailty Index)**			
	**YES**	**NO**	
**Mobility.**			
Can the patient perform the following tasks without assistance from another person (walking aids such as a can or a wheelchair are allowed)			
1. Grocery shopping	0	1	
2. Walk outside house (around house or to neighbour)	0	1	
3. Getting (un)dressed	0	1	
4. Visiting restroom	0	1	
**Vision**			
5. Does the patient encounter problems in daily life because of impaired vision?	1	0	
**Hearing**			
6. Does the patient encounter problems in daily life because of impaired hearing?	1	0	
**Nutrition**			
7. Has the patient unintentionally lost a lot of weight in the past 6 months (6kg in 6 months or 3kg in 3 months)?	1	0	
**Co-morbidity**			
8. Does the patient use 4 or more different types of medication?	1	0	
**YES**	**NO**	**SOMETIMES**	
**Cognition**			
9. Does the patient have any complaints on his/her memory (or diagnosed with dementia)?	1	0	0
**Psychosocial**			
10. Does the patient ever experience emptiness around him?	1	0	1
e.g. You feel so sad that you have no interest in your surroundings. Or if someone you love no longer love you, how do you feel?			
11. Does the patient ever miss the presence of other people around him? *Or do you miss anyone you love?*	1	0	1
12. Does the patient ever feel left alone?	1	0	1
e.g. You wish there is someone to go with you for something important.			
13. Has the patient been feeling down or depressed lately?	1	0	1
14. Has the patient felt nervous or anxious lately?	1	0	1
**Physical Fitness**			
15. How would the patient rate his/her own physical fitness?	1	0	
(0-10 ; 0 is very bad, 10 is very good)			
0 – 6 = 1 and 7 – 10 = 0			

Supplement 2)

G8 score			
	**Items**	**Score**	**Possible answers**
**A**	Has food intake declined over the past 3 months due to loss of appetite, digestive problems, chewing or swallowing difficulties?	0	severe decrease in food intake
		1	moderate decrease in food intake
		2	no decrease in food intake
**B**	Weight loss during the last 3 months	0	weight loss > 3 kg
		1	does not know
		2	weight loss between 1 and 3 kgs
		3	no weight loss
**C**	Mobility	0	bed or chair bound
		1	able to get out of bed/chair but does not go out
		2	goes out
**E**	Neuropsychological problems	0	severe dementia or depression
		1	mild dementia or depression
		2	no psychological problems
**F**	Body Mass Index (BMI (weight in kg) / (height in m^2)	0	BMI < 19
		1	BMI = 19 to BMI < 21
		2	BMI = 21 to BMI < 23
		3	BMI = 23 and > 23
**H**	Takes more than 3 medications per day	0	yes
		1	no
**P**	In comparison with other people of the same age, how does the patient consider his/her health status?	0	not as good
		0.5	does not know
		1	as good
		2	better
	Age	0	>85
		1	80-85
		2	<80
